# Oligometastatic head and neck cancer: Which patients benefit from radical local treatment of all tumour sites?

**DOI:** 10.1186/s13014-021-01790-w

**Published:** 2021-03-31

**Authors:** Thomas Weissmann, Daniel Höfler, Markus Hecht, Sabine Semrau, Marlen Haderlein, Irina Filimonova, Benjamin Frey, Christoph Bert, Sebastian Lettmaier, Konstantinos Mantsopoulos, Heinrich Iro, Rainer Fietkau, Florian Putz

**Affiliations:** 1 Department of Radiotherapy, Friedrich-Alexander-Universität Erlangen-Nürnberg, Universitaetsstraße 27, 91054 Erlangen, Germany; 2 Department of Otolaryngology, Head and Neck Surgery, Friedrich-Alexander-Universität Erlangen-Nürnberg, Erlangen, Germany

**Keywords:** Oligometastatic disease, Local treatment, Radiotherapy, SBRT, Head and neck cancer

## Abstract

**Background:**

There is a large lack of evidence for optimal treatment in oligometastatic head and neck cancer and it is especially unclear which patients benefit from radical local treatment of all tumour sites.

**Methods:**

40 patients with newly diagnosed oligometastatic head and neck cancer received radical local treatment of all tumour sites from 14.02.2008 to 24.08.2018. Primary endpoint was overall survival. Time to occurrence of new distant metastases and local control were evaluated as secondary endpoints as well as prognostic factors in univariate und multivariate Cox’s regression analysis. To investigate the impact of total tumour volume on survival, all tumour sites were segmented on baseline imaging.

**Results:**

Radical local treatment included radiotherapy in 90% of patients, surgery in 25% and radiofrequency ablation in 3%. Median overall survival from first diagnosis of oligometastatic disease was 23.0 months, 2-year survival was 48%, 3-year survival was 37%, 4-year survival was 24% and 5-year survival was 16%. Median time to occurrence of new distant metastases was 11.6 months with freedom from new metastases showing a tail pattern after 3 years of follow-up (22% at 3, 4- and 5-years post-treatment). In multivariate analysis, better ECOG status, absence of bone and brain metastases and lower total tumour volume were significantly associated with improved survival, whereas the number of metastases and involved organ sites was not.

**Conclusions:**

Radical local treatment in oligometastatic head and neck cancer shows promising outcomes and needs to be further pursued. Patients with good performance status, absence of brain and bone metastases and low total tumour volume were identified as optimal candidates for radical local treatment in oligometastatic head and neck cancer and should be considered for selection in future prospective trials.

**Supplementary Information:**

The online version contains supplementary material available at 10.1186/s13014-021-01790-w.

## Background

Oligometastatic disease is an increasingly recognized disease entity in metastatic solid malignancies characterized by limited metastatic burden and consecutive benefit of local treatment to all metastatic sites [1–3]. Introduced at a conceptual level as early as 1995 by Hellman and Weichselbaum [4], it was in very recent years that prospective randomized Phase II trials increasingly provided empirical evidence for improved outcome with radical local treatment to all tumour sites in cohorts consisting mainly of oligometastatic non-small cell lung cancer (NSCLC) and prostate cancer patients [5–8]. While these Phase II trials by design were not able to provide confirmatory evidence, they strongly indicated a systemic effect of local treatment. Among other studies, the well-recognized Phase II trial by Gomez et al., reported significantly improved overall survival (41.2 vs. 17 months) as well as decreased occurrence of new distant lesions in oligometastatic NSCLC patients [5]. Encouraged by such promising results, scientific societies like the EORTC, ESTRO and ASTRO have put forth diagnostic criteria as well as classification proposals for oligometastatic disease in recent months [1, 2]. Despite these efforts, oligometastatic disease remains poorly defined and the most widely recognized criterion of oligometastatic disease is the ability to safely apply local treatment to all tumour sites, which is largely because of a great lack of scientific studies to base any additional criteria upon. As such it is currently still largely unclear, which patients should receive radical local treatment of all metastatic sites in addition to or instead of systemic treatment for metastatic disease. This is especially true in metastatic head and neck cancers, in which the significance and potential benefit of radical local treatment remains largely unexplored to this date and only very few and small series have been published so far. Although investigation of the oligometastatic paradigm is particularly challenging in metastatic head and neck cancers, any resulting contribution to current systemic treatment options for patients with metastatic disease could be of particular value.

Starting from 2008 and as a multi-disciplinary endeavour, patients with metastatic head and neck cancers received local treatment to all tumour sites as part of routine clinical care at the University Hospital Erlangen, as long as safe application of local treatment to all locations was ensured. In the present work we report the clinical results and treatment details of this cohort. Furthermore, we explore potential prognosticators including tumour volume-based metrics that have been implicated in the definition of oligometastatic disease to further elucidate which patients with metastatic head and neck cancer will benefit the most from radical local treatment of all tumour sites.

## Methods

### Patient population

Patients with metastatic head and neck cancer received ablative local treatment to all tumour sites at the University hospital Erlangen as part of routine clinical care. Patients were selected for radical local treatment, if all tumour sites could be safely treated locally, but no formal thresholds e.g., for number of metastases or involved organ sites was used. This unique setting enabled us to investigate prognostic factors that could improve patient selection and optimize the definition of oligometastatic disease in head and neck cancers. For this retrospective analysis we identified forty head and neck cancer patients who had been first diagnosed with synchronous or metachronous metastases and subsequently received radical local treatment of all tumour sites irrespective of the number of metastases or involved organ sites. According to the recent ESTRO/EORTC classification, 68% (27/40) of these patients suffered from metachronous oligorecurrence, whereas 33% (13/40) had synchronous oligometastatic disease [2]. 30% (12/40) had an active locoregional tumour manifestation in addition to distant metastases. 55% (22/40) of patients had histologic proof of metastatic disease, while 45% (18/40) had imaging diagnosis of metastases alone.

### Treatment

Local treatments were recommended after joint interdisciplinary review by experts in radiation oncology, head and neck surgery, interventional radiology as well as thoracic and visceral surgery within the framework of an interdisciplinary tumour board with recommendations being based on patient- and disease-specific as well as technical considerations with the aim to achieve local ablation of each tumour site in the safest possible manner. In total 90% (36/40) of patients received radiotherapy as part of their treatment for oligometastatic disease (OMD), 25% (10/40) of patients received surgery and 1 patient (3%) received radiofrequency ablation of liver metastases. A detailed description of local treatments by individual patient case is provided in Additional file 1: Table S1. Patients routinely received restaging using computed tomography 6 weeks after local treatment and then subsequently at intervals of 3 months.

Regarding systemic treatment, 35% (14/40) of patients received platinum-based combination treatment, 20% (8/40) received single agent cytostatic chemotherapy or cetuximab alone, 5% (2/40) were treated with cetuximab + platinum combination therapy and 5% (2/40) received immune checkpoint inhibitor treatment. Systemic treatment was deferred in 14 patients with a solitary metastasis, who received imaging follow-up at close intervals following local treatment of metastatic disease (Table 1).

**Table 1:** Patient characteristics at first local treatment

Parameter	Total cohort (N = 40)
ESTRO/EORTC Type of oligometastatic disease, n (%)	
Metachronous Oligorecurrence	27 (68%)
Synchronous Oligometastatic disease	13 (33%)
Age, years	
Median (IQR)	60.5 (56.3–70.8)
Mean (range)	62.0 (41.0–82.0)
ECOG, n (%)	
ECOG 0	5 (13%)
ECOG 1	18 (45%)
ECOG 2	14 (35%)
ECOG 3	3 (8%)
Histology, n (%)	
Squamous cell carcinoma	33 (83%)
Lymphoepithelial carcinoma	3 (8%)
Adenocarcinoma	2 (5%)
Neuroendocrine carcinoma	1 (3%)
Undifferentiated	1 (3%)
Original site of Head and Neck primary, n (%)	
Hypopharynx	11 (28%)
Larynx	9 (23%)
Oropharynx	7 (18%)
Head and neck cancer of unknown primary	5 (13%)
Nasal cavity/paranasal sinuses	4 (10%)
Oral cavity	3 (8%)
Nasopharynx	1 (3%)
Number of metastases	
Median (IQR)	1.0 (1.0–2.0)
Mean (range)	1.6 (1.0–7.0)
Metastatically involved organ systems, n (%)	
One organ system	34 (85%)
Two organ systems	6 (15%)
Metastatically involved organ sites^a^, n (%)	
Pulmonary	23 (58%)
Lymphonodal	11 (28%)
Bone	6 (15%)
Hepatic	3 (8%)
Brain	2 (5%)
Total tumor volume, cm^3^	
Median (IQR)	19.7 (2.0-46.8)
Mean (range)	49.5 (0.2–550.8)
Total metastases volume, cm^3^	
Median (IQR)	9.4 (1.6–23.2)
Mean (range)	24.5 (0.2–240.6)
Number of active tumor sites (metastases + primary)	
Median (IQR)	2.0 (1.0–2.0)
Mean (range)	1.8 (1.0–7.0)
Locoregional tumor, n (%)	
No	28 (70%)
Primary manifestation	7 (18%)
Recurrence	5 (13%)
Locoregional tumor site, n (%)	
Primary and involved regional lymph nodes	7 (18%)
Regional lymph nodes only	3 (8%)
Primary only	2 (5%)
Total locoregional tumor volume, cm^3^	
Median (IQR)	0.0 (0.0–7.0)
Mean (range)	24.9 (0.0–502.4)
Previous head and neck radiotherapy, n (%)	
Prior head and neck radiotherapy	31 (78%)
No prior head and neck radiotherapy	9 (23%)
Previous head and neck surgery, n (%)	
Prior head and neck surgery	23 (58%)
No prior head and neck surgery	17 (43%)
Histologic proof of metastatic disease, n (%)	
Yes	22 (55%)
No	18 (45%)
FDG-PET staging, n (%)	
Yes	13 (33%)
No	27 (68%)
Interval from diagnosis of OMD to first local treatment, months	
Median (IQR)	1.3 (0.9–2.2)
Mean (range)	1.7 (0–7.3)
Total duration of OMD first-line treatment, months	
Median (IQR)	3.1 (2.2–4.6)
Mean (range)	3.7 (0.3–10.8)
Local treatment for OMD^a^, n (%)	
Radiotherapy	36 (90%)
Surgery	10 (25%)
Interventional radiology	1 (3%)
Biologically effective dose (α/β = 10)^b^, Gy	
Median (IQR)	78.8 (67.2–111.4)
Mean (range)	82.6 (39.0–115.2)
Systemic treatment, n (%)	
Platinum-combination treatment	14 (35%)
Single-agent cytostatic chemotherapy alone	4 (10%)
Cetuximab alone	4 (10%)
Platinum-combination + Cetuximab	2 (5%)
Immune checkpoint inhibitor	2 (5%)
No concurrent systemic treatment	14 (35%)

*OMD* oligometastatic disease

^a^Some patients are part of multiple categories

^b^Minimum biologically effective dose to tumor locations in patients that received radiotherapy

### Volumetric analysis

In baseline imaging all tumour sites were semiautomatically segmented in every patient using the NVIDIA Clara AI-assisted annotation extension for the OpenSource software 3D Slicer v.4.11.0 [9], which is a neural-network based autosegmentation solution. All autosegmentations were then manually validated and corrected by an experienced radiation oncologist. Total mesh-based tumour volumes were calculated from these segmentations using SlicerRadiomics [10]. 3D Renderings and slice-based representations were created using 3DSlicer v.4.11.0.

### Statistical analysis

Primary endpoint was overall survival. Time to occurrence of new distant metastases and local control were evaluated as secondary endpoints as well as prognostic factors in univariate und multivariate Cox’s regression analysis.

The number of metastases and involved organ sites was counted in baseline imaging with multiple lymph node metastases in one lymph node region (e.g., multiple mediastinal lymph nodes) being counted as one distant metastasis. Overall survival was calculated from the first diagnosis of oligometastatic disease, i.e., date of first imaging showing metastases, until death or censored at last follow-up. Time to new distant metastases was similarly determined from first diagnosis of oligometastatic disease to occurrence of new distant metastases or censored at death or last follow-up. Local control was calculated at a lesion-level from the date of first local treatment for oligometastatic disease until progression according to RECIST 1.1 criteria [11] or censored at last follow-up or death. Time to event outcomes were assessed using the Kaplan–Meier method and the logrank test. Prognostic factors were first assessed using univariate Cox’s regression analysis and significant prognosticators (*p* < 0.05) from univariate analysis were then included in the multivariate Cox’s regression model. Statistics were calculated using SPSS 21.0, Graphs were created using GraphPad Prism 8.4.

## Results

**Fig. 1:**
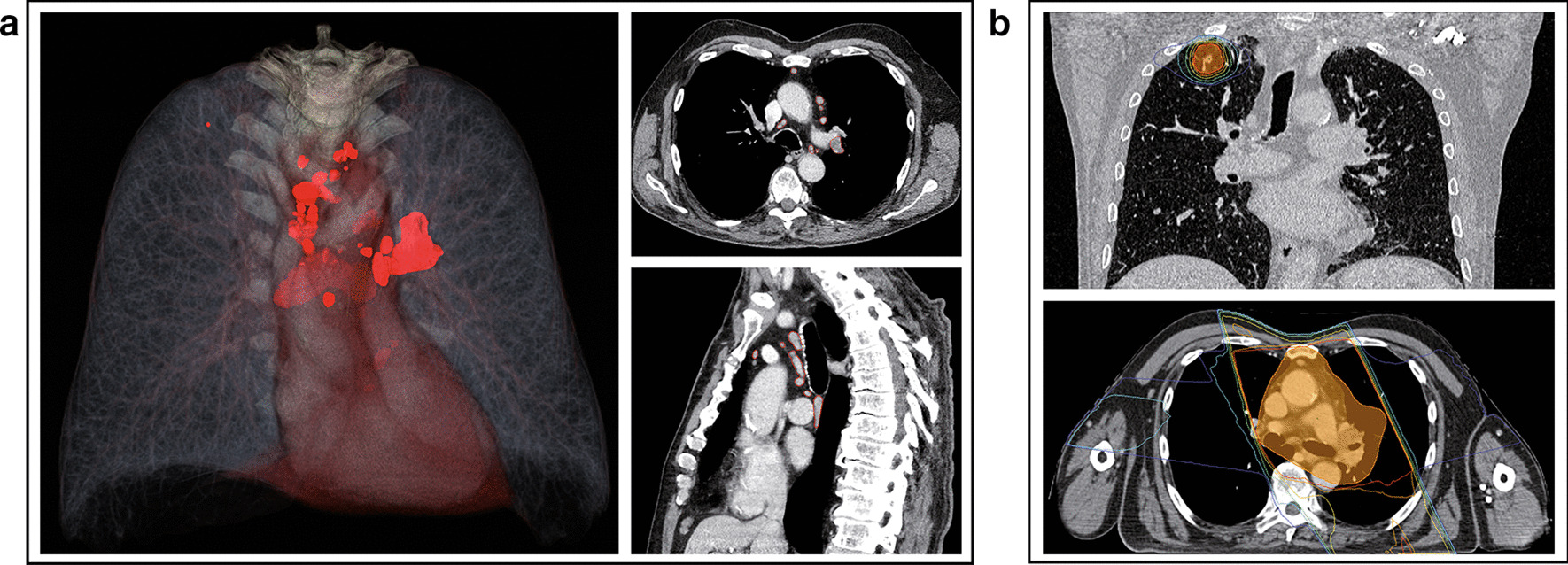
Total tumour volume (**a**) and corresponding radiotherapy treatment plans (**b**) in a patient with mediastinal lymph node metastases and a single pulmonary lesion from metastatic laryngeal cancer. **a**: All tumour sites were analysed volumetrically via tumour segmentations in all patients to obtain total tumour volumes at diagnosis of oligometastatic disease. Left: 3D rendering showing segmented mediastinal lymph node metastases and a single right-upper lobe metastasis (red). Right: Axial and sagittal view of segmented lymph node metastases. **b**: Radiotherapy treatment plan showing isodoses (red: 95%, orange: 90%, yellow: 80%, green: 60%, cyan: 40% and blue: 30%) and planning target volumes of stereotactic body radiotherapy to the right-upper lobe metastasis (12 × 6 Gy) as well as of conventionally fractionated chemoradiation (25 × 1.8 Gy + 12 × 1.8 Gy Boost [not shown]) of mediastinal lymph node metastases

A total of 40 patients with newly diagnosed oligometastatic head and neck cancer received ablative local treatment to all tumour sites. Median age was 60.5 years (range, 41–82 years). Median number of metastases was 1 (range, 1–7) with the lung being the most commonly affected organ site (58%). 30% of patients (12/40) had active locoregional tumour in addition to distant metastases. The median interval from first imaging diagnosis of oligometastatic disease (OMD) to start of first local treatment (i.e., first local treatment of metastases or locoregional tumour manifestations) was 1.3 months. The median duration to subsequently complete all local treatments was 3.1 months. 90% (36/40) of patients received radiotherapy as part of their treatment for OMD, 25% (10/40) of patients received surgery and 1 patient (3%) received radiofrequency ablation of liver metastases. 73% (29/40) of patients were treated exclusively with radiotherapy for OMD. Full details concerning cohort characteristics and treatment schedules are listed in Table 1 and Additional file 1: Table S1. Total tumour volume was determined for all patients via segmentation of all tumour sites in imaging studies at the time of first diagnosis of OMD (Fig. 1).

**Fig. 2:**
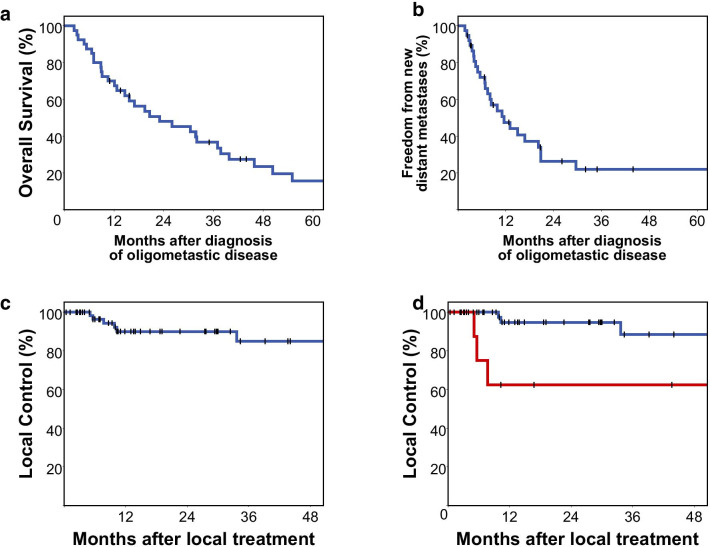
**a** Kaplan–Meier plot showing overall survival since first diagnosis of oligometastatic disease and **b** freedom from new distant metastases in all 40 patients. **c** Local control in all of the 75 treated tumour sites and **d** local control for locoregional tumour manifestations versus distant metastases

After a median follow-up of 65.2 months 83% (33/40) of patients had died. Median overall survival from first diagnosis of oligometastatic disease was 23.0 months. 1-year overall survival was 70%, 2-year survival was 48%, 3-year survival was 37%, 4-year survival was 24% and 5-year survival was 16% (Fig. 2a). In the subgroup of patients that were exclusively treated with radiotherapy (n = 29), median overall survival was 20.6 months. 1-year overall survival was 69%, 2-year survival was 45%, 3-year survival was 37%, 4-year survival was 23% and 5-year survival was 15%.

Median time to occurrence of new distant metastases was 11.6 months with 1-year freedom from distant metastases being 48%, 2-year freedom from new distant metastases being 26% and 3-year freedom from new distant metastases being 22%. Interestingly, freedom from new distant metastases subsequently ceased to decline and showed a tail pattern with 22% of patients remaining free from new distant metastases at 4- and 5-years post diagnosis of oligometastatic disease (Fig. 2b). Similar findings were obtained in the subgroup exclusively treated with radiotherapy. In patients treated exclusively with radiotherapy, median time to occurrence of new distant metastases was 9.9 months with 1-year freedom from distant metastases being 45%, 2-year freedom from new distant metastases being 35% and 3-year, 4-year and 5-year freedom from new distant metastases being 29%.

Regarding local treatment effect, 8 out of a total of 75 lesions showed progression after a median imaging follow-up of 19.1 months. 1-year and 2-year local control was 90%, 3-year and 4-year local control was 85%. There was no difference between treatment modalities (logrank *p* = 0.324). Local control was higher for metastases than for locoregional head and neck tumour manifestations without reaching significance, however (1-year local control 95% vs. 63%, *p* = 0.122, Fig. 2c, d). These locoregional tumour manifestations were primary tumours in 58% (7/12) and recurrent disease in 42% (5/12). Radical local treatment of locoregional tumour manifestations was surgery alone in 25% (3/12), chemoradiation in 58% (7/12) and surgery followed by chemoradiation in 17% (2/12). All treatments and tumour sites are reported in full detail in Additional file 1: Table S1 at an individual patient level.

**Fig. 3:**
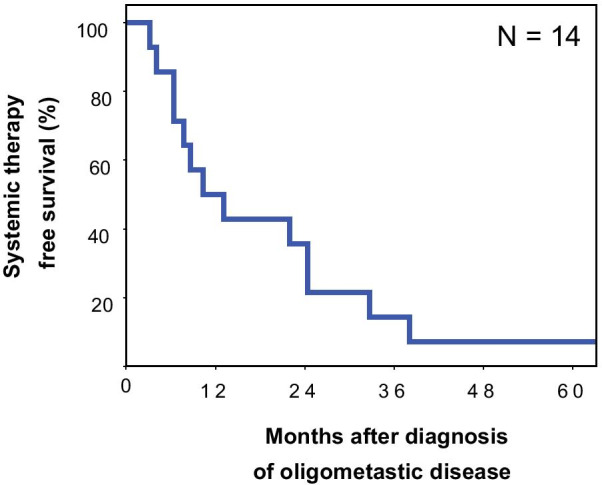
Systemic therapy-free survival in the subgroup of patients, in which systemic therapy was deferred after local treatment of metastases (N = 14)

Despite numerically worse local control for locoregional manifestations, overall survival was not significantly different for patients with and without active locoregional tumour manifestations (*p* = 0.574, see Table 2 and below). In a subgroup of 35% (14/40) patients with solitary metastasis, systemic treatment was deferred after local treatment. 79% (11/14) of these patients had pulmonary metastasis. In this subgroup without initial systemic treatment, 1-year systemic treatment-free survival was 50% with 2- and 3-year systemic treatment-free survival being 36% and 14%, respectively (Fig. 3).

**Table 2:** Univariate Cox’s regression analysis of prognostic factors for overall survival (N = 40)

Parameter	Univariate	
	HR (95% CI)	*p* value
ECOG, per point	2.8 (1.6–4.7)	< 0.001
Bone metastases, yes versus no	7.3 (2.6–20.5)	< 0.001
Brain metastases, yes versus no	12.5 (2.5–69.6)	0.004
Total tumor volume, per 100 cm^3^	1.6 (1.2–2.1)	0.005
Pulmonary metastases, yes versus no	0.4 (0.2–0.8)	0.011
Locoregional tumor volume, per 100 cm^3^	1.5 (1.0–2.2)	0.029
Total metastases volume, per 100 cm^3^	2.5 (1.1–5.7)	0.031
Radiotherapy dose, BED10 per 10 Gy	0.8 (0.7–1.0)	0.052
Number of involved organ systems	0.4 (0.1–1.3)	0.110
Time interval from OMD diagnosis to first local treatment, months	0.8 (0.6––1.1)	0.170
Number of metastases	0.8 (0.6–1.1)	0.189
Systemic treatment, yes versus no	1.6 (0.8–3.3)	0.229
Cetuximab, yes versus no	1.7 (0.6–4.5)	0.298
Distant nodal metastases, yes versus no	0.7 (0.3–1.6)	0.341
PET Staging	0.7 (0.3–1.6)	0.398
Hepatic metastases, yes versus no	0.6 (0.2–2.0)	0.407
Duration of local treatments for OMD, months	0.9 (0.8–1.1)	0.454
Age, per 10 years	1.2 (0.8–1.7)	0.460
Metachronous versus synchronous OMD	0.8 (0.4–1.6)	0.502
Checkpoint inhibitor treatment, yes versus no	1.6 (0.4–6.6)	0.553
Active locoregional tumor manifestation	1.2 (0.6–2.6)	0.574
Platin combination chemotherapy, yes versus no	0.9 (0.5–2.0)	0.859

To determine which patients with metastatic disease benefit from radical local treatment to all tumour sites, a large number of potential prognosticators was investigated (Table 2). In univariate analysis worse ECOG score (HR 2.8 per point, *p* < 0.001), the presence of bony (HR = 7.3, *p* < 0.001) and brain metastases (HR 12.5, *p* = 0.004), higher total tumour volume (HR 1.6 per 100 cm^3^, *p* = 0.005) as well as higher locoregional tumour volume (HR 1.5 per 100 cm^3^, *p* = 0.029) and higher total metastases volume (HR 2.5, *p* = 0.031) were significantly associated with worse survival. In contrast the presence of pulmonary metastases (HR = 0.4, *p* = 0.011) was significantly associated with improved survival and higher radiotherapy dose (HR 0.8 per 10 Gy BED10, *p* = 0.052) showed a trend towards significantly improved prognosis. Conversely neither the number of metastases (*p* = 0.189), nor the number of involved organ sites (*p* = 0.110) and age (*p* = 0.460) were significant prognosticators in the present cohort. Similarly, there was no significant difference in survival between patients with synchronous oligometastatic disease and metachronous oligorecurrence (*p* = 0.502) (Table 2).

**Fig. 4:**
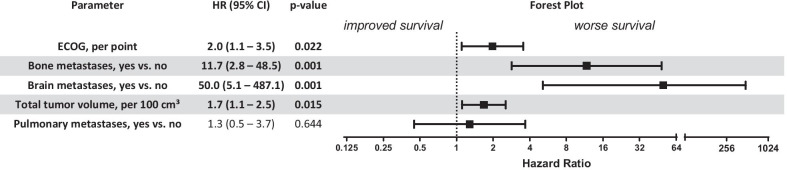
Multivariate Cox’s regression analysis and Forrest plot of prognostic factors for overall survival following radical local treatment of all tumour sites

In multivariate analysis for overall survival, better ECOG status, lower total tumour volume and the absence of brain as well as bony metastases remained significant predictors of improved survival following radical local treatment of all tumour sites (Fig. 4).

## Discussion

Treatment outcomes in metastatic head and neck cancer treated with current systemic treatment options remain unsatisfactory. Platinum-based chemotherapy plus cetuximab achieved a median overall survival of 10.1 months in the well-known study by Vermorken et al. [12]. More recently the addition of pembrolizumab to platinum and 5-FU significantly improved overall survival in the total population of the KEYNOTE-048 trial over cetuximab with platinum and 5-FU (13.0 vs. 10.7 months). In the subset of patients with a PD-L1 combined positive score (CPS) of ≥ 1 and ≥ 20 the benefit of pembrolizumab + chemotherapy was more pronounced, achieving a median overall survival of 13.6 and 14.7 months, respectively [13]. Considering that treatments in our study occurred mostly before the introduction of immune checkpoint inhibitors into routine clinical practice, the median overall survival of 23.0 months achieved in the present study with an increased fraction of patients alive after 2 years has to be considered as a clear indication that the oligometastatic treatment paradigm holds promise in metastatic head and neck cancer. While selection effects cannot be fully discarded in a retrospective setting, it has to be noted, that over half of patients treated in the Phase III trial by Vermorken et al. suffered from locoregionally recurrent tumour only while all patients in the present cohort had metastatic disease. Similarly, only up to 12% of patient in the Vermorken trial had a Karnofsky score of less than 80% while 43% of patients in the present cohort were characterized by an ECOG score of 2 or worse (i.e., Karnofsky score of < 80%) [12].

Very few series on oligometastatic head and neck cancer have been reported so far in the literature. Schulz et al. reported on a cohort of 37 patients with metastatic head and neck cancer, in which distant metastases were treated specifically with either surgery or stereotactic body radiotherapy (SBRT) [14]. Observing a median overall survival of 23.97 months, their outcome was remarkably similar to the one achieved in our series. Similarly, Bates et al. reported a median overall survival of 22.8 months in a cohort of patients with oligometastatic head and neck cancer (≤ 5 metastases) treated with SBRT. Therefore, published series on oligometastatic head and neck cancer show a very consistent median overall survival of 23–24 months. Series that are limited exclusively to patients with pulmonary oligometastases form a notable exception to this rule, however. Bonomo et al. for instance achieved a median overall survival of 47 months in a cohort of patients with metastatic head and neck cancer limited to the lungs [15] and Pasalic similarly achieved a median overall survival of around 48 months (value obtained from Kaplan–Meier plot) in a cohort of metastatic head and neck cancer patients with up to 3 lung-only metastases treated with SBRT to all tumour sites [16]. The improvement in overall survival that is consistently observed with local treatment of oligometastatic disease indicates that local treatments may affect systemic disease progression in patients with metastatic cancer. The fact that freedom from new distant metastases ceased to decline after 3 years with 22% of patients remaining free from new distant metastases at 4- and 5-years post diagnosis of oligometastatic disease in the present series is an interesting observation in this regard. Resembling the finding of decreased distant metastasis formation in NSCLC patients in the Phase II study by Gomez et al., a similar systemic impact of local treatment could also mediate improvements in overall survival in patients with oligometastatic head and neck cancer [5].

An important aim of the present study was to identify prognostic factors for improved survival with radical local treatment and to determine which patients with metastatic head and neck cancer should receive local treatment of all tumour sites. The exploration of prognostic parameters in oligometastatic head and neck cancers treated in radical intent at all tumour sites had only been partially addressed by previous studies. As such, Bates et al. investigated several prognostic factors in a cohort of 27 radically treated patients including number of metastases and involved organ sites but did not find pre-treatment factors that were significantly associated with overall survival [17]. Schulz et al. among others investigated prognostic factors in large cohorts of patient with metastatic head and neck cancers with and without local treatment to all tumour sites [14]. While, importantly all these studies were able to show the prognostic advantage of local treatment and limited metastatic burden, predictors of outcome of patients that actually received radical local treatment was outside the scope of previous series. In the present study, we explored a large number of potential prognosticators first in univariate analysis and included significant parameters subsequently in the final multivariate model. In univariate analysis, we found worse ECOG score and the presence of bone and brain metastases to be negative predictors of overall survival following radical local treatment of all tumour sites in metastatic head and neck cancer. Conversely and in line with previous series, the presence of pulmonary metastases was associated with improved survival in univariate analysis. Moreover, we explored the impact of volume-based metrics on overall survival in oligometastatic head and neck cancer. Total tumour volume and other volume-related parameters have frequently been hypothesized as potential selection criteria for the definition of oligometastatic disease but had not been investigated in oligometastatic head and neck cancer so far [1, 2]. We used an AI-based autosegmentation approach to segment all tumour sites on baseline imaging for calculation of tumour volumes in every patient. Interestingly, total tumour volume was in fact strongly associated with survival in oligometastatic head and neck cancer. In multivariate analysis, lower total tumour volume, better ECOG status and the absence of brain and bony metastases remained significant predictors for improved survival following radical local treatment of all tumour sites. These results could help in identifying patients with metastatic head and neck cancer who benefit from an oligometastatic treatment paradigm and should be further evaluated in future studies.

A particularly interesting scientific question is whether local treatment could be used to delay initiation of systemic treatment in oligometastatic disease. In our cohort, systemic treatment had been deferred in a subset of 35% of patients consisting mainly of patients with solitary lung metastasis. In this subgroup, 1-year systemic treatment-free survival was 50% with 2- and 3-year systemic treatment-free survival being 36% and 14%, respectively. In a retrospective series on lung oligometastases from different primaries, Mazzola et al. showed that lung SBRT achieved a median systemic treatment-free survival of 16 months [18]. In the setting of oligorecurrent prostate cancer, the well-known randomized Phase II STOMP trial by Ost et al. demonstrated a significantly longer androgen deprivation therapy-free survival for metastasis-directed therapy versus observation [7]. Also, in a large multicentre retrospective study by Triggiani et al., the authors demonstrated a promising 1-year systemic treatment-free survival of 72.1% in oligoprogressive castration-resistant prostate cancer [19]. Collectively, these results indicate a systemic effect of local treatment in oligometastatic disease, and that metastasis-directed therapy is able to substantially delay the initiation of systemic treatment in these patients. However, whether the combination of local treatment with upfront systemic treatment for oligometastatic disease provides additional benefit over delayed administration and in which patients systemic treatment can be safely deferred needs additional prospective and randomized trials to be answered definitely.

While patients in the present series were largely treated before the current era of checkpoint inhibitors, advances in immunotherapies are an important consideration for future studies on oligometastatic head and neck cancer. As synergistic effects for the combination of radiotherapy and checkpoint inhibitor treatment have been described [20–22], the synthesis of local treatment and systemic immunotherapy could be especially beneficial in the oligometastatic setting. Ongoing trails like IMPORTANCE (NCT03386357) and CheckRad-CD8 (NCT03426657) are already investigating optimal combination strategies of radiotherapy and checkpoint inhibitor treatment and results are eagerly awaited.

### Limitations

Being a retrospective study hidden selection effects could have influenced results. The small patient number was a limitation that precluded detailed subgroup analyses and resulted in reduced statistical power.

## Conclusions

Radical local treatment in oligometastatic head and neck cancer showed promising outcomes in this study and clearly warrants further research. Patients with favourable performance status, absence of brain and bone metastases and low total tumour volume were identified as optimal candidates for radical local treatment in oligometastatic head and neck cancer and should be considered for selection in future prospective trials.

## Supplementary Information

**Additional file 1:** Detailed listing of treatment for de-novo oligometastatic disease and corresponding tumor locations in all patients.

## Data Availability

The raw data supporting the conclusions of this article will be made available by the authors, without undue reservation.
