# Supplementary material for: Oligometastatic head and neck cancer: Which patients benefit from radical local treatment of all tumour sites?
Source: Radiat Oncol. 2021 Mar 31;16:62. doi: 10.1186/s13014-021-01790-w (PMC8011153; doi:10.1186/s13014-021-01790-w)
Supplement: Supplementary file 1 — Additional file 1: Detailed listing of treatment for de-novo oligometastatic disease and corresponding tumor locations in all patients. [file 13014_2021_1790_MOESM1_ESM.docx]

**Detailed listing of treatment for de-novo oligometastatic disease
and corresponding tumor locations in all patients**

| **Patient** | **Site of Disease** | **Treatment** |
| --- | --- | --- |
| **#1** |  |  |
|  | 1) Locoregional (Local recurrence Epiglottis) | Concurrent chemobrachytherapy (47.6 Gy in 14 fractions, concurrent Cisplatin, and interstitial hyperthermia) |
|  | 2) Pulmonary (Right middle lobe) | HFRT (60 Gy in 20 fractions) |
| **#2** |  |  |
|  | 1) Lymphonodal (Mediastinum) | RCT (56 Gy in 28 fractions, concurrent weekly Paclitaxel) |
|  | 2) Bone (Right Os ilium) | RCT (50 Gy in 25 fractions, concurrent weekly Paclitaxel) |
| **#3** |  |  |
|  | 1) Locoregional (Primary   + Cervical lymph nodes) | Resection + adjuvant chemoradiation (R0 resection and unilateral, modified radical neck dissection, 64 Gy in 32 fractions, concurrent 5-FU/Cisplatin) |
|  | 2) Lymphonodal (Mediastinum + Left hilum) | RCT (66 Gy in 33 fractions, concurrent 5-FU/Cisplatin) |
| **#4** |  |  |
|  | 1) Bone (Base of skull) | Resection + definitive chemoradiation (Partial resection, 45 Gy in 25 fractions, concurrent 5-FU/Cisplatin) |
| **#5** |  |  |
|  | 1) Pulmonary (Right lower lobe) | SABR (72 Gy in 12 fractions) |
|  | 2) Pulmonary (Left upper lobe) | HFRT (60 Gy in 20 fractions) |
| **#6** |  |  |
|  | 1) Pulmonary (Right middle lobe) | SABR (72 Gy in 12 fractions) |
| **#7** |  |  |
|  | 1) Bone (Base of skull) | RCT (39 Gy in 13 fractions, concurrent Paclitaxel/Carboplatin) |
| **#8** |  |  |
|  | 1) Locoregional (Primary + Cervical lymph nodes) | RCT (72 Gy in 36 fractions, concurrent 5-FU/Cisplatin) |
|  | 2) Pulmonary (Left upper lobe) | SABR (72 Gy in 12 fractions) |
| **#9** |  |  |
|  | 1) Pulmonary (Right upper lobe) | SABR (48 Gy in 12 fractions) |
| **#10** |  |  |
|  | 1) Pulmonary (Left lower lobe) | SABR (72 Gy in 12 fractions) |
| **#11** |  |  |
|  | 1) Pulmonary (Left upper lobe) | SABR (50 Gy in 5 fractions) |
|  | 2) Pulmonary (Right lower lobe) | SABR (50 Gy in 5 fractions) |
|  | 3) Pulmonary (Right upper lobe) | SABR (50 Gy in 5 fractions) |
| **#12** |  |  |
|  | 1) Brain (Solitary brain metastasis) | SRS (1 x 20 Gy) |
|  | 2) Locoregional (Local recurrence) | Resection (R0) |
| **#13** |  |  |
|  | 1) Lymphonodal (Mediastinum) | RCT (66.6 Gy in 37 fractions, concurrent Carboplatin) |
| **#14** |  |  |
|  | 1) Lymphonodal (Mediastinum) | Resection (Right lower lobectomy and mediastinal lymph node dissection) |
|  | 2) Pulmonary (Superior right lower lobe) | Resection (Right lower lobectomy and mediastinal lymph node dissection) |
|  | 3) Pulmonary (Anterior right lower lobe) | Resection (Right lower lobectomy and mediastinal lymph node dissection) |
|  | 4) Pulmonary (Inferior right lower lobe) | Resection (Right lower lobectomy and mediastinal lymph node dissection) |
| **#15** |  |  |
|  | 1) Pulmonary (Right middle lobe) | SABR (48 Gy in 12 fractions) |
|  | 2) Pulmonary (Right upper lobe) | SABR (50 Gy in 5 fractions) |
| **#16** |  |  |
|  | 1) Pulmonary (Right upper lobe) | HFRT (60 Gy in 15 fractions) |
| **#17** |  |  |
|  | 1) Bone (Base of skull) | RCT (45 Gy in 25 fractions, concurrent Carboplatin) |
| **#18** |  |  |
|  | 1) Bone (left upper thoracic aperture) | RCT (30 Gy in 10 fractions, concurrent Cisplatin/Paclitaxel) |
|  |  |  |
| **Patient** | **Site of Disease** | **Treatment** |
| **#19** |  |  |
|  | 1) Pulmonary (Right upper lobe) | Resection (Wedge resection) |
| **#20** |  |  |
|  | 1) Brain (Solitary brain metastasis) | Partial resection and postoperative FSRT (41.4 Gy in 23 fractions) |
| **#21** |  |  |
|  | 1) Hepatic (Solitary hepatic metastasis segment VI) | Resection (Hepatic segmentectomy) |
|  | 2) Locoregional (Cervical lymph node recurrence) | Resection (Right neck dissection) |
| **#22** |  |  |
|  | 1) Pulmonary (Right upper lobe) | SABR (72 Gy in 12 fractions) |
|  | 2) Lymphonodal (Mediastinum) | RCT (66.6 Gy in 37 fractions, concurrent Cisplatin) |
| **#23** |  |  |
|  | 1) Pulmonary (Posterior right lower lobe) | SABR (72 Gy in 12 fractions) |
|  | 2) Pulmonary (Central right lower lobe) | SABR (72 Gy in 12 fractions) |
| **#24** |  |  |
|  | 1) Locoregional (Right cervical lymph node) | Resection + adjuvant chemoradiation (Right cervical neck dissection, 64 Gy in 32 fractions, concurrent 5-FU/Cisplatin) |
|  | 2) Lymphonodal (Mediastinum) | RCT (70 Gy in 35 fractions, concurrent 5-FU/Cisplatin) |
| **#25** |  |  |
|  | 1) Hepatic (Segment 2/3) | RFA (CT-guided) |
|  | 2) Hepatic (Segment 8/5) | RFA (CT-guided) |
|  | 3) Hepatic (Segment 6) | RFA (CT-guided) |
|  | 4) Pulmonary (Left upper lobe) | SABR (72 Gy in 12 fractions) |
| **#26** |  |  |
|  | 1) Lymphonodal (Mediastinum) | RCT (66.6 Gy in 37 fractions, concurrent Carboplatin/Vinorelbin) |
| **#27** |  |  |
|  | 1) Pulmonary (Left lower lobe) | SABR (72 Gy in 12 fractions) |
|  | 2) Locoregional (Cervical lymph node recurrence) | Resection (Right neck dissection) |
| **#28** |  |  |
|  | 1) Lymphonodal (Mediastinum) | RCT (66.6 Gy in 37 fractions, concurrent Cisplatin/5-FU) |
|  | 2) Locoregional (Primary and cervical nodal metastases) | RCT (70 Gy in 35 fractions, concurrent Cisplatin/Paclitaxel) |
| **#29** |  |  |
|  | 1) Pulmonary (Right upper lobe) | SABR (72 Gy in 12 fractions) |
| **#30** |  |  |
|  | 1) Locoregional (Primary and cervical nodal metastases) | RCT (72 Gy in 36 fractions, concurrent Carboplatin) |
|  | 2) Lymphonodal (Mediastinum) | RCT (66 Gy in 33 fractions, concurrent Carboplatin) |
| **#31** |  |  |
|  | 1) Pulmonary (Left upper lobe) | SABR (72 Gy in 12 fractions) |
| **#32** |  |  |
|  | 1) Lymphonodal (Right axilla) | RAT (59.4 Gy in 33 fractions, concurrent Cetuximab) |
| **#33** |  |  |
|  | 1) Pulmonary (Right upper lobe) | SABR (72 Gy in 12 fractions) |
|  | 2) Lymphonodal (Mediastinum) | RCT (66.6 in 37 fractions, concurrent Cisplatin/Paclitaxel) |
| **#34** |  |  |
|  | 1) Pulmonary (#1 left) | Resection (Pulmonary wedge resection) |
|  | 2) Pulmonary (#2 left) | Resection (Pulmonary wedge resection) |
|  | 3) Pulmonary (#3 left) | Resection (Pulmonary wedge resection) |
|  | 4) Pulmonary (#4 left) | Resection (Pulmonary wedge resection) |
|  | 5) Pulmonary (#5 left) | Resection (Pulmonary wedge resection) |
|  | 6) Pulmonary (#1 right) | Resection (Pulmonary wedge resection) |
|  | 7) Pulmonary (#2 right) | Resection (Pulmonary wedge resection) |
| **#35** |  |  |
|  | 1) Pulmonary (Left upper lobe) | SABR (72 Gy in 12 fractions) |
|  | 2) Pulmonary (Right lower lobe) | SABR (72 Gy in 12 fractions) |
|  | 3) Pulmonary (Left lower lobe) | SABR (72 Gy in 12 fractions) |
|  |  |  |
|  |  |  |
| **Patient** | **Site of Disease** | **Treatment** |
| **#36** |  |  |
|  | 1) Locoregional (Primary and cervical nodal  metastases) | RCT (72 Gy in 36 fractions, concurrent Cisplatin/5-FU) |
|  | 2) Lymphonodal (Mediastinum) | RCT (66.6 Gy in 37 fractions, concurrent Cisplatin/5-FU) |
|  | 3) Pulmonary (Right upper lobe) | SABR (72 Gy in 12 fractions) |
| **#37** |  |  |
|  | 1) Locoregional (Primary and cervical nodal  metastases) | RAT (70 Gy in 35 fractions, concurrent Cetuximab) |
|  | 2) Hepatic (Solitary hepatic metastasis) | SABR (72 Gy in 12 fractions) |
| **#38** |  |  |
|  | 1) Locoregional (Primary and cervical nodal  metastases) | RCT (72 Gy in 36 fractions, concurrent Carboplatin/5-FU) |
|  | 2) Bone (Os ilium right) | SABR (48 Gy in 12 fractions) |
| **#39** |  |  |
|  | 1) Pulmonary (Right upper lobe) | HFRT (60 Gy in 15 fractions) |
| **#40** |  |  |
|  | 1) Pulmonary (Left lower lobe) | SABR (72 Gy in 12 fractions) |

HFRT = Hypofractionated radiotherapy, RCT = Radiochemotherapy, SABR = Stereotactic ablative body radiotherapy, RFA = Radiofrequency ablation
